# Supplementary material for: DNA supercoiling-mediated G4/R-loop formation tunes transcription by controlling the access of RNA polymerase
Source: Nat Commun. 2025 Apr 9;16:3363. doi: 10.1038/s41467-025-58479-x (PMC11982182; doi:10.1038/s41467-025-58479-x)
Supplement: Supplementary file 1 — Supplementary Information [file 41467_2025_58479_MOESM1_ESM.pdf]

SUPPLEMENTARY INFORMATION

DNA supercoiling-mediated G4/R-loop formation tunes transcription by controlling the access of RNA polymerase

Jihee Hwang[†], Chun-Ying Lee[†], Tapas Paul, Huijin Lee, Alanna Craig, Taekjip Ha and Sua Myong[*]

Supplementary document

Modeling suggests supercoiling absorption by R-loops drives transcription suppression.

We utilized a previously developed simulation model of transcription that incorporates the interplay between DNA topology and RNAP translocation [Tripathi 2022] and implemented G4 and R-loop states (Supplementary Fig. 14). After recruitment and an initial phase of rotation, RNAPs, bulky with the attached mRNA, tend to deposit positive (negative) DNA supercoils downstream (upstream). In case of a plasmid, as implemented here, the positive supercoils (positive torque) built up at the end of the gene cancel the negative supercoils (negative torque) at the promoter proximal region. However, the cancellation is not instantaneous, we implement a plectoneme relaxation event that equilibrates the torque, that allows non-equilibrium buildup of DNA torsion between relaxation events. We use a transcription initiation or RNAP recruitment rate that is depends on the promoter supercoiling status, as observed experimentally (Fig. 5g and Supplementary Fig. 14). The model has a transient R-loop state with a nucleation rate equal to the transcription initiation rate and a very fast off-rate (1 per second), hence transient. Negatively supercoiled plasmid promotes transcription initiation that also increases the nucleation of transient R-loops. This transient R-loop state, featuring perturbed B-DNA, acts a precursor to the G4 and stable R-loop states. Once a stable R-loop is nucleated, it absorbs DNA linking number and reduces the supercoiling density in the rest of the plasmid, especially the promoter region. The G4 state acts as a facilitator for the stable R-loop state. Once a G4 is nucleated for a PQS, the transient R-loop is trapped in an ON-state, facilitating stable R-loop nucleation. Using a G4 nucleation rate (2 per min) that is faster than that of the stable R-loop (1 per min), we find that the simulations recapitulated the experimental trend of transcription suppression for PQS (Fig. 7c and Supplementary Fig. 14). The supercoiling density of the promoter gradually becomes less negative due to higher probabilities of G4 and R-loop (Fig. 7d and Supplementary Figs. 14 and 15). Additionallly, the simulations rationalize the reduced G4/R-loop probabilities upon Topo 1 treatment (Fig. 6e), as a result of the decreased basal supercoiling density in the plasmid (Supplementary Fig 14).

	NT-sp30					NT-control				
NTP	-	-	+	+	+	-	-	+	+	+
S9.6 (ug)	0	20	0	6.6	20	0	20	0	6.6	20
lane	1	2	3	4	5	6	7	8	9	10

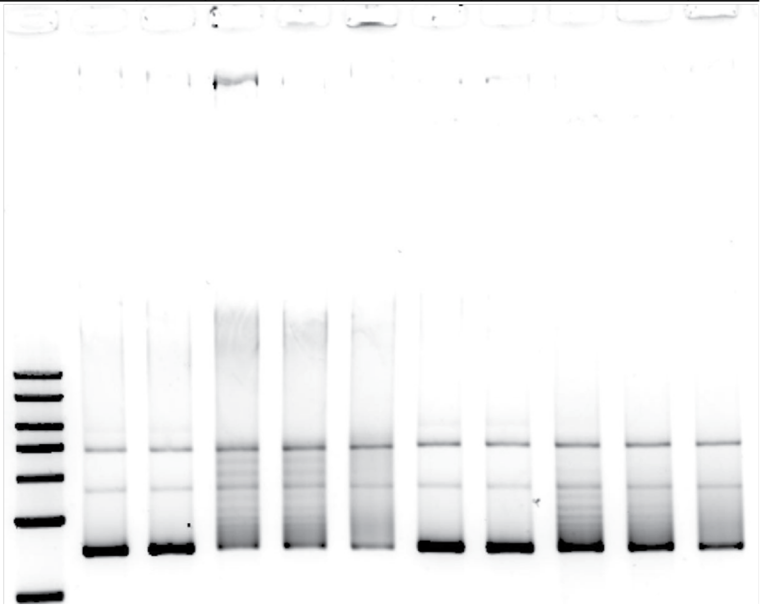

Supplementary Fig. 1: The shifted bands are composed of R-loops.

S9.6, a monoclonal antibody that specifically binds to R-loops, is premixed with the transcribed samples of either NT-sp30 or the Control. The premixtures are separated on 1% agarose gel. As increasing S9.6, the shifted multiple bands are disappeared (comparing lane 3 to 5, and lane 8 to10) and accumulated at the gel well. This result indicates that the shifted bands are composed of R-loops. The extent of band disappearance is different as NT-sp30 and Control have different levels of R-loop-mediated topological relaxation.

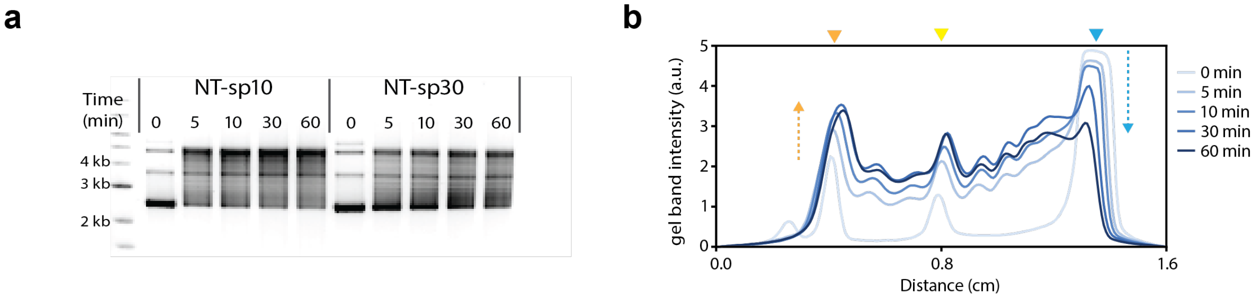

Supplementary Fig. 2: The kinetics of R-loop formation in NT-sp10 and NT-sp30

(a) The gel image of Figure 2e.(b) The band distribution of NT-sp30.

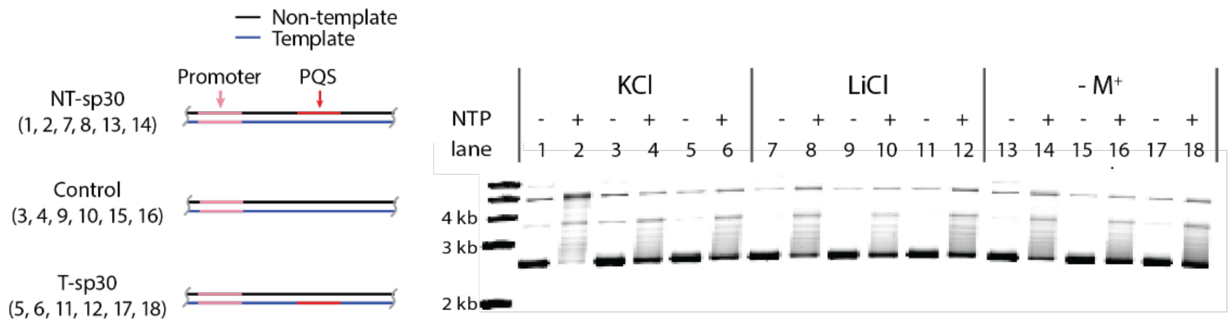

Supplementary Fig. 3: G4 stability affects R-loop-mediated topological relaxation

G4 stability is affected by mono-valent cations in the order of K+ > Li+ > No monovalent metal cation. To investigate the contribution of G4 to R-loop-mediated topological changes, *in vitro* transcription is performed using either KCl, LiCl, or no mono-valent cation, and the transcribed samples are then separated on a 1% agarose gel. NT-sp30 leads to reduced topological relaxation according to the G4 stability in the order of K+ > Li+ > No monovalent metal cation. However, Control or T-sp30 didn't show significant differences according to monovalent metal cation. These results support that G4 assists R-loop-mediated topological relaxation by stabilizing R-loops as expected[1,2].

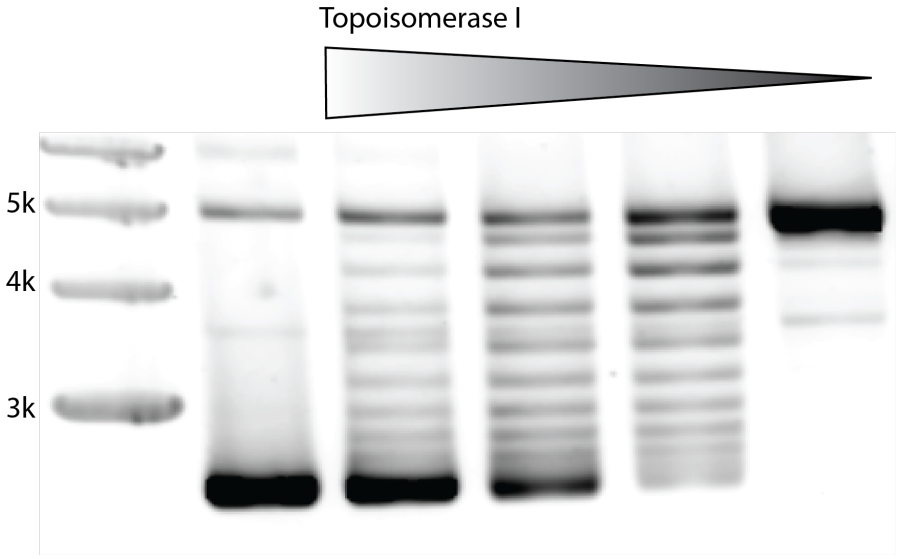

Supplementary Fig. 4: Topoisomerase 1 (Topo 1) pre-treated NT-sp10

To investigate the impact of initial supercoiling status of DNA construct on G4/R-loop-mediated transcriptional suppression, Topoisomerases 1 is pre-treated to generate less supercoiled DNA. The first lane represents the initial supercoiling status of plasmid, purified from e.coli. 5 ug of each plasmid is pre-treated with 2.5 (lane 2), 3.32 (lane 3), 5 (lane 4) and 10 units (lane 5) of *E.coli* Topo 1. As increasing the concentration of Topo 1, the bands shift toward the top band which is the most relaxed status.

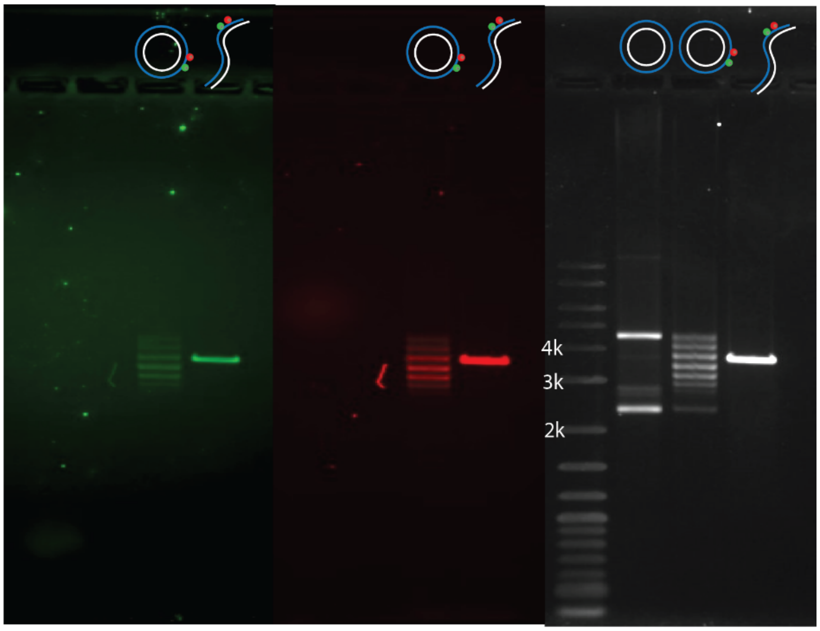

Supplementary Fig. 5: The gel image of linearized [FRET1]

First two gels are imaged by exciting Cy3 and Cy5, respectively. The third gel is post-stained with SYBR green 2. The linearized [FRET1] was prepared by treating the relaxed [FRET1] with BamHI.

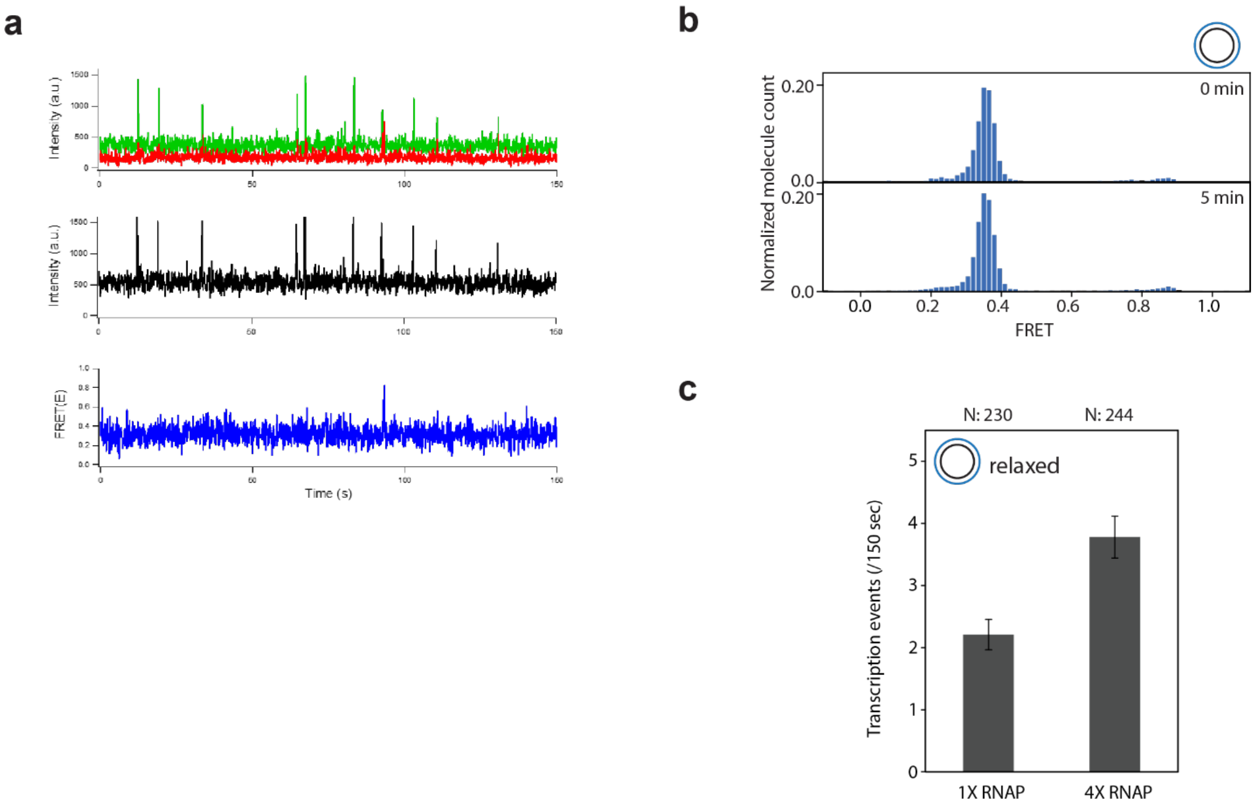

Supplementary Fig. 6: Concentration-dependent RNAP turnover rate

(a) Representative single-molecule time traces of 4X RNAP transcription for relaxed PQS construct. (b) FRET histograms of relaxed [FRET1] before (0 min) and after 5 min transcription (5 min) (c) The quantification of PIFE peaks for 1X (1.5%) and 4X RNAP 7.5%). In relaxed DNA, the frequency of PIFE peaks indicates a turnover rate that varies with RNAP concentration.

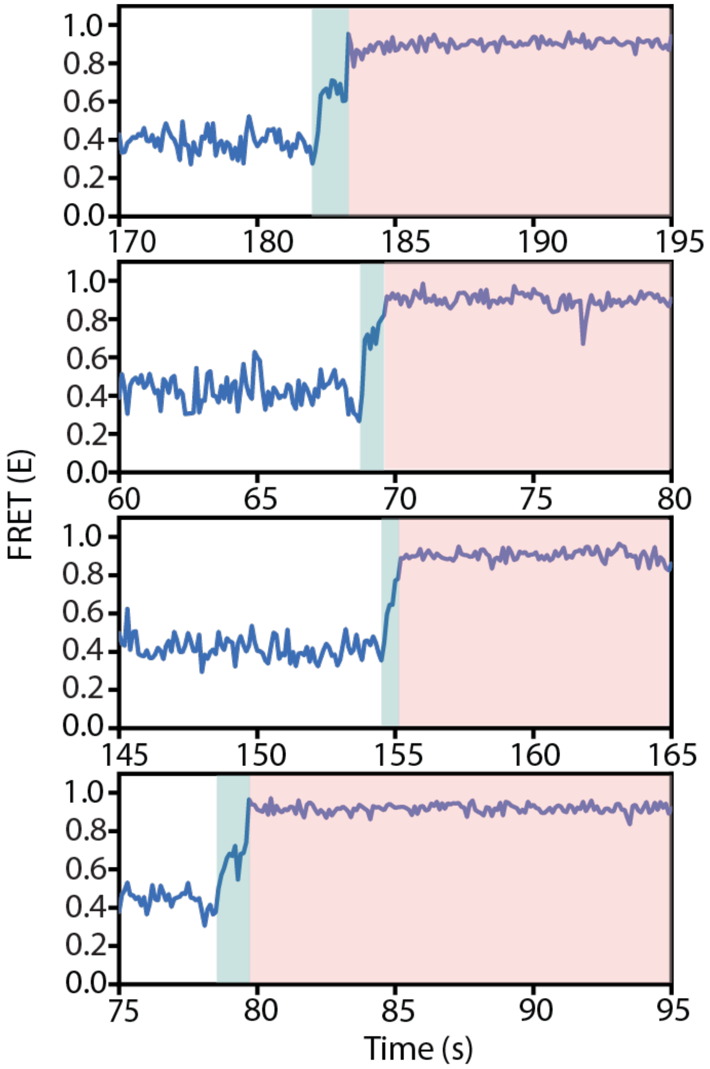

Supplementary Fig. 7: Stepwise FRET transition from E~0.7 to E~0.9 of supercoiled [FRET1]-NT-PQS

Representative time-traces of supercoiled [FRET1]-NT-PQS showing a stepwise FRET transition from E~0.7 to E~0.9. 97.5 % of DNA folded into G4 (E~0.9) (119/122) exhibited the stepwise FRET transition. 2.5 % of DNA (3/122) shows an immediate transition from 0.4 to 0.9 without the intermediate state (E~0.7). It is supposed to be a limitation of the camera's read speed (100 ms).

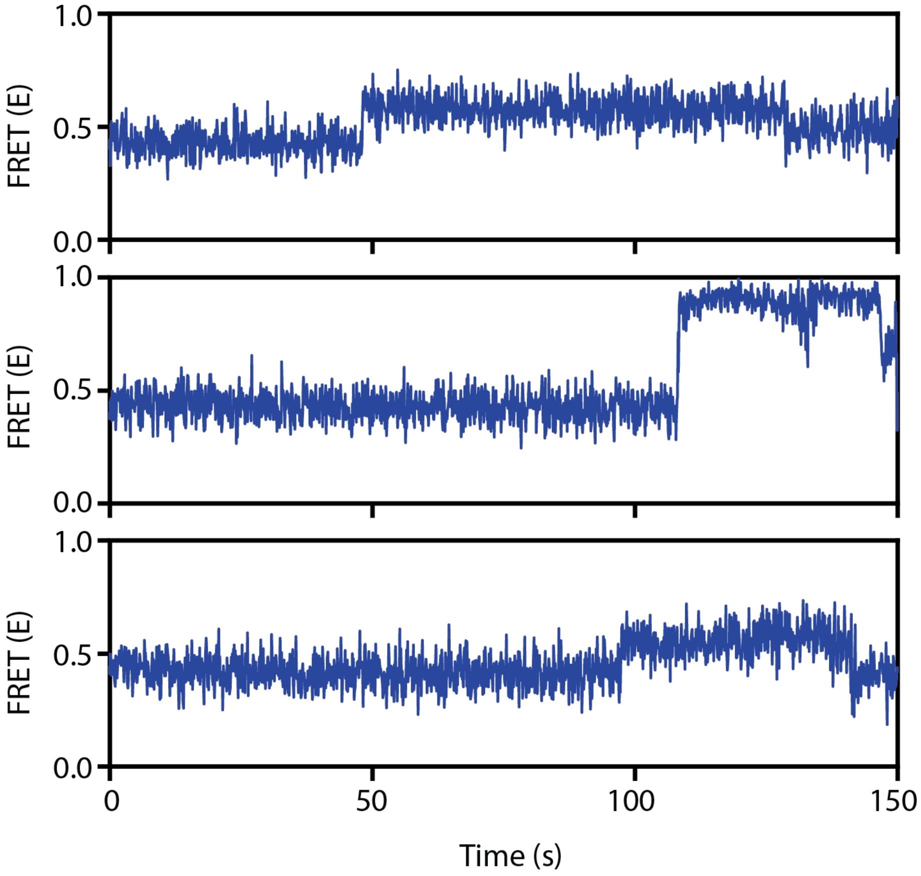

Supplementary Fig. 8: Representative FRET traces of linearized [FRET1] with NT-PQS

Representative FRET traces of linearized [FRET1] with NT-PQS. The linearized DNA showed a long-lived R-loop infrequently (3/138) with very slow kinetics.

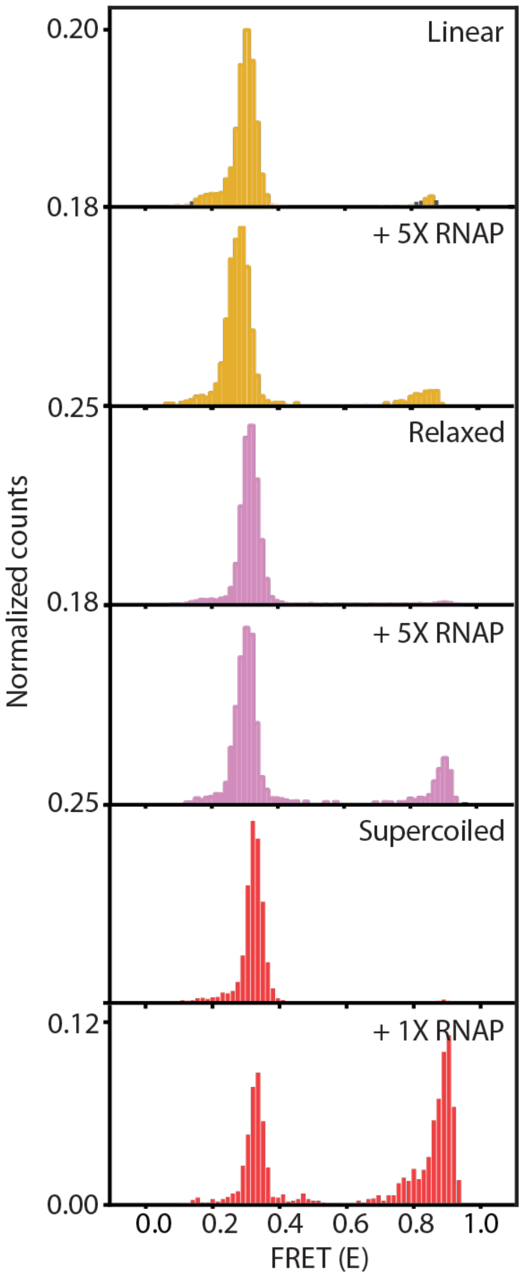

Supplementary Fig. 9: Histograms of G4/R-loop formation affected by DNA superhelicity and RNAP concentration

To examine the effect of DNA superhelicity and RNAP concentration, we carried out transcription reactions on the slide for 30 minutes and measured by TIRF. From the top, the histogram shows linear [FRET1]-NT-PQS before and after transcription with 5X RNAP (Yellow), relaxed [FRET1]-NT-PQS before and after transcription with 5X RNAP (purple), and supercoiled [FRET1]-NT-PQS before and after transcription with 1X RNAP (red). The fraction of high FRET is considered as G4/R-loop formation. The middle FRET is not considered because the fraction is negligible compared to the High FRET.

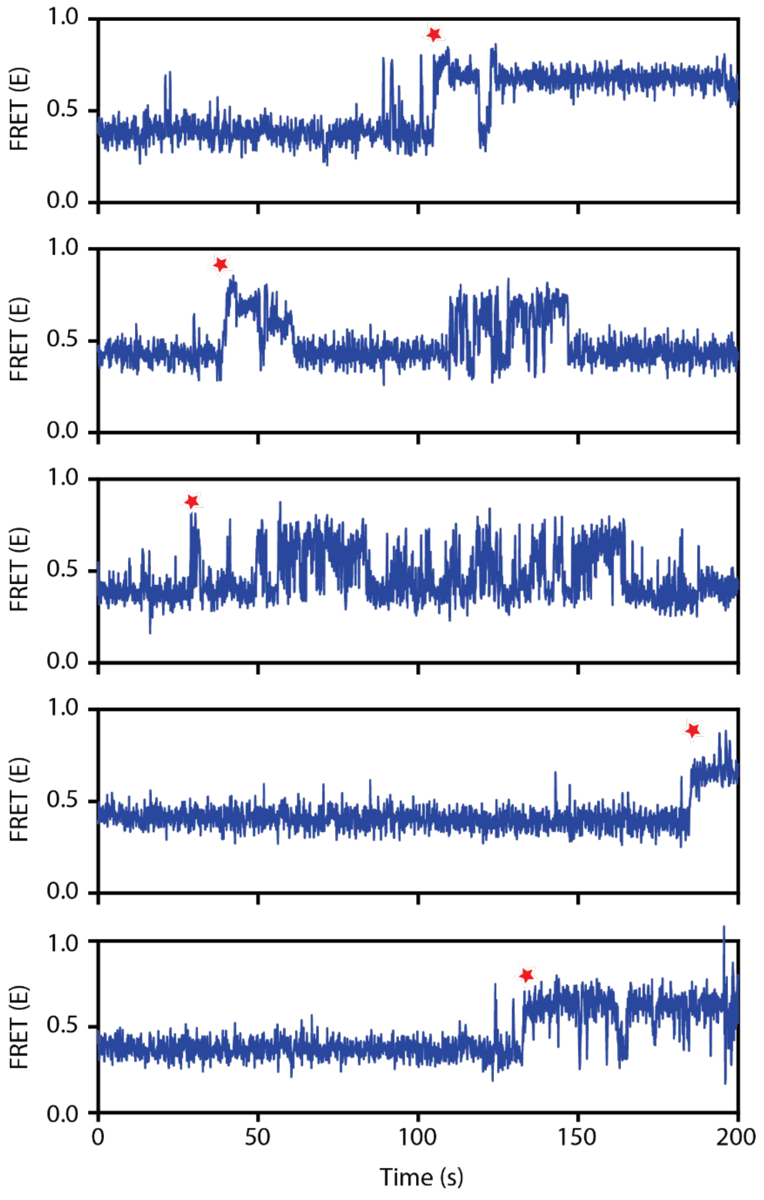

Supplementary Fig. 10: Representative FRET traces of supercoiled Control [FRET1]

Individual FRET traces of supercoiled Control [FRET1] for Figure 6d. The red star marks the synchronized time for Figure 6d, bottom.

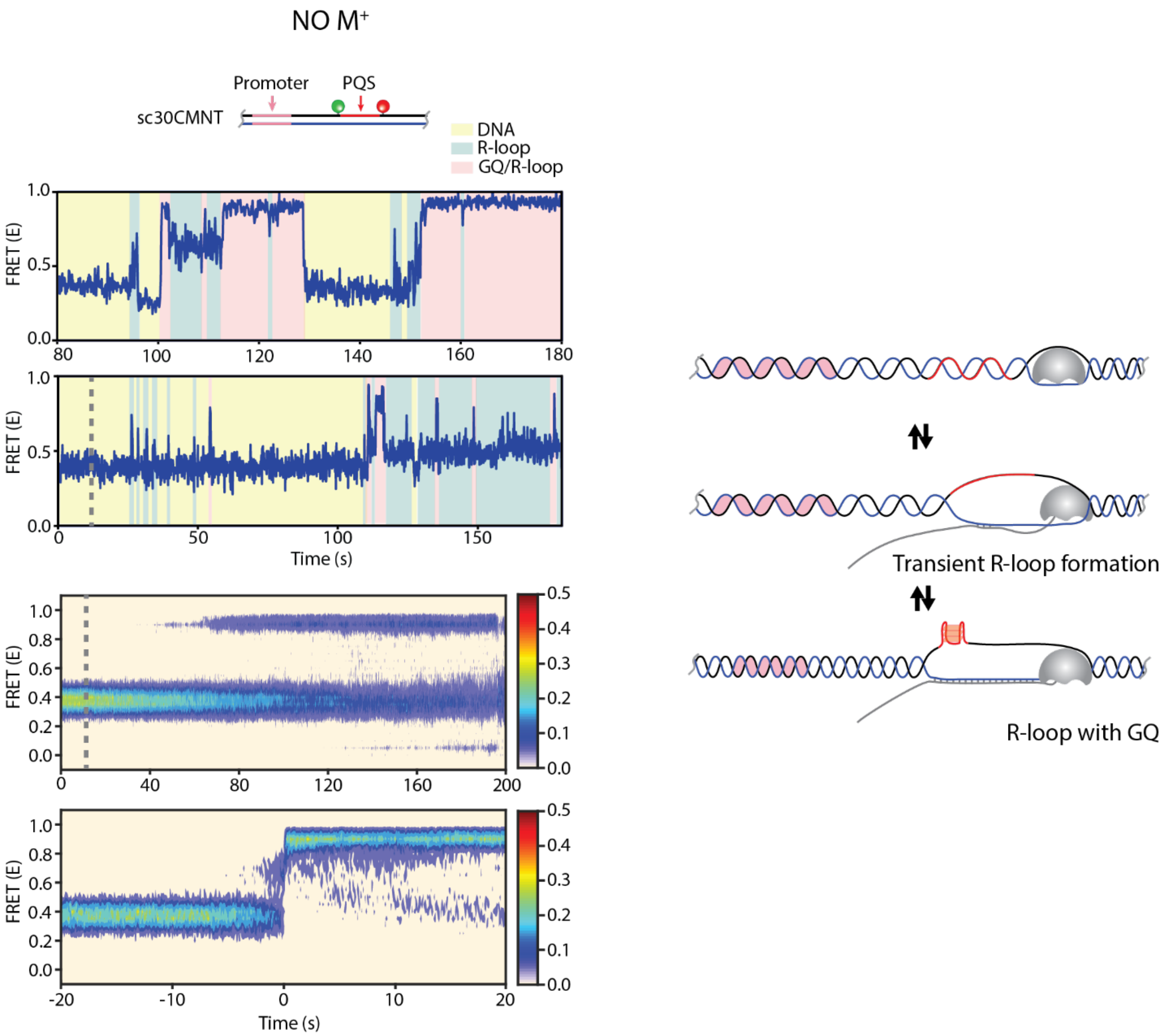

Supplementary Fig. 11: supercoiled [FRET1] with NT-PQS in G4 destabilizing condition.

Representative FRET trace and heat map of supercoiled [FRET1] with NT-PQS in G4 destabilizing condition (-M^+, no mono-valent ion). It forms a long-lived high FRET (~0.9. G4), but it showed a fluctuating transition between love-lived R-loop and G4, which is similar behavior with supercoiled [FRET1] with Control in KCl.

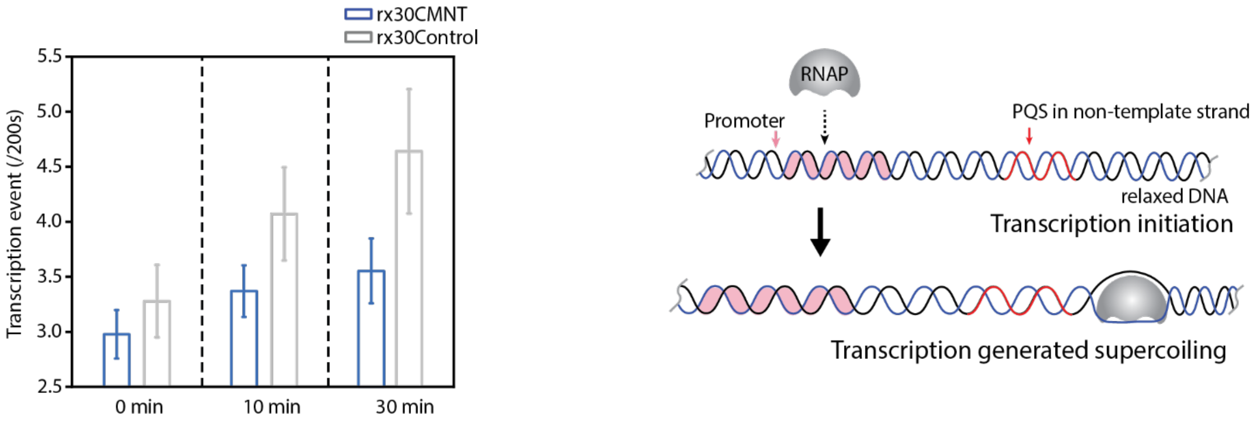

Supplementary Fig. 12: Transcription-generated negative supercoiling enhances the transcription rate by making the promoter region more accessible in the relaxed construct.

The left panel quantifies the number of FRET peaks observed within 200 seconds after RNAP injection at time points 0 min, 10 min, and 30 min for NT-PQS and Control constructs (left y-axis, shown in blue and gray bars, respectively) in relaxed [FRET2]. Data are based on 3-4 replicates with over 200 molecules. The right schematic illustrates how multiple rounds of transcription increase RNAP loading rate by making the promoter more accessible. In the presence of G4 formation, NT-PQS may relieve negative supercoiling upon forming G4/R-loop structures.

	lnNT-sp10			lnNT-sp30		
NTP	-	+	+	-	+	+
DNase I	-	+	+	-	+	+
RNase A	-	-	+	-	-	+
lane	1	2	3	4	5	6

	scNT-sp10			scNT-sp30		
NTP	-	+	+	-	+	+
DNase I	-	+	+	-	+	+
RNase A	-	-	+	-	-	+
lane	7	8	9	10	11	12

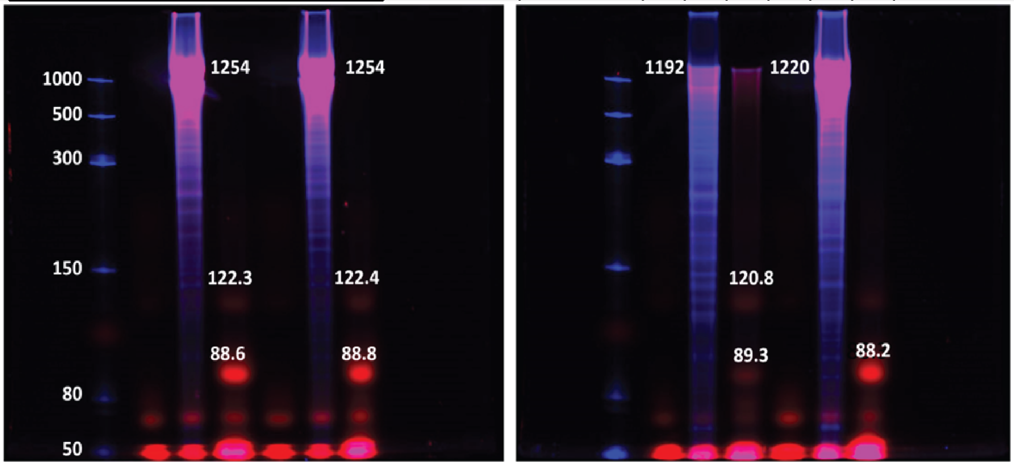

Supplementary Fig. 13: Measurement of R-loop sizes of linear and supercoiled DNA with sp10 and sp30

To evaluate the size of R-loops in linear and supercoiled DNA with NT-sp10 and -sp30, 1 uM Cy5-UTP was introduced into the transcription reaction. DNase I and RNase A were then applied to digest the DNA and RNA outside of R-loops, leaving only the RNA contained within the R-loops intact. The size of the R-loop was determined by calibrating against the single-stranded RNA ladder.

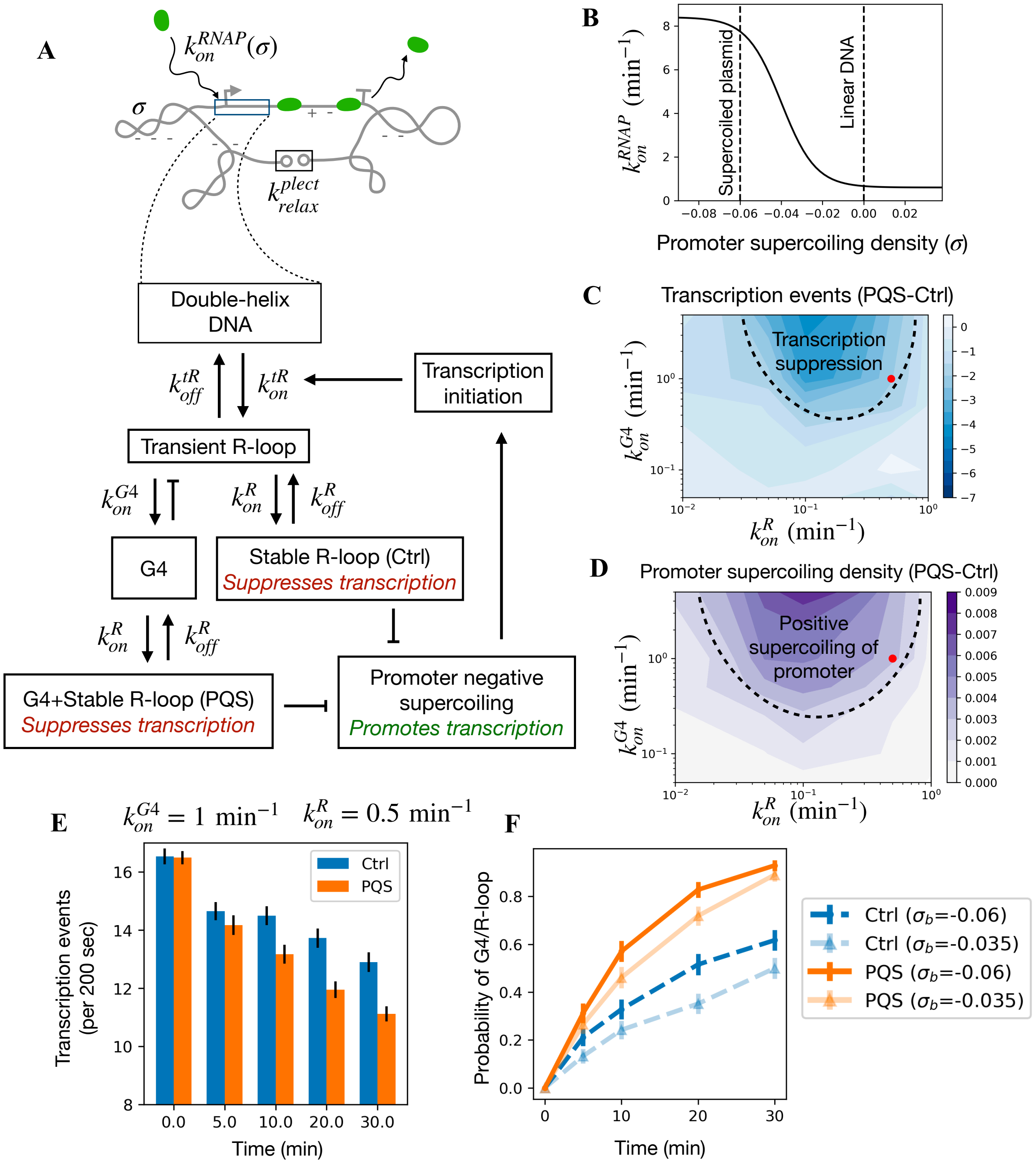

Supplementary Fig. 14: Simulation model of transcription in a plasmid with G4/R-loop nucleation.

(A) Model schematic showing the supercoiled plasmid with the gene. The different structural states of the promoter-proximal DNA and their transition rates are shown. The transient R-loop is formed when RNAP passes through the promoter proximal region. This transient state facilitates the G4 state in presence of PQS, which then freezes the transient R-loop into an ON state. The transient R-loop also leads to the stable R-loop state which acts as a torsional defect and absorbs DNA linking number, thus driving a decrease in promoter supercoiling, eventually lowering

transcription initiation. Additionally, there is a plectoneme relaxation event occurring at 30 per min, that equilibrates the torque in the non-transcribed segment connecting the transcription initiation site to the termination site. (B) The variation of the transcription initiation rate with promoter supercoiling density, where the dashed lines correspond to the experimentally observed initiation rates for supercoiled and linear DNA (See Fig. 5g). (C) Regime diagram with the stable R-loop rate along x-axis and G4 formation rate along y-axis. The color shows the difference in transcription initiation events recorded over a 200-sec window 30 min after transcription induction for PQS and Ctrl sequences. When G4 rates are higher than the stable R-loop rates, there is suppression of transcription (dark blue color). (D) Regime diagram with same axes as in (C) where the purple color shows the change in the promoter supercoiling status between PQS and Ctrl. The transcription suppression regime in (C) corresponds to relative positive supercoiling of the promoter. (E) Transcription events recorded over a 200-sec window at various time points post transcription induction. Here, we used the G4 formation rate as 1 per min and stable R-loop rate is 0.5 per min, corresponding to the red circle in (C) and (D) (Compare with Fig. 7c). (F) Temporal evolution of the probabilities of the G4+R-loop state for PQS and the stable R-loop state for Ctrl. The probabilities show a slower increase when the basal supercoiling density of the plasmid is less negative. We hypothesize that treatment with Topo 1 lowers the negative supercoiling density of the plasmid leading to lower G4/R-loop formation (compare with Fig. 6e).

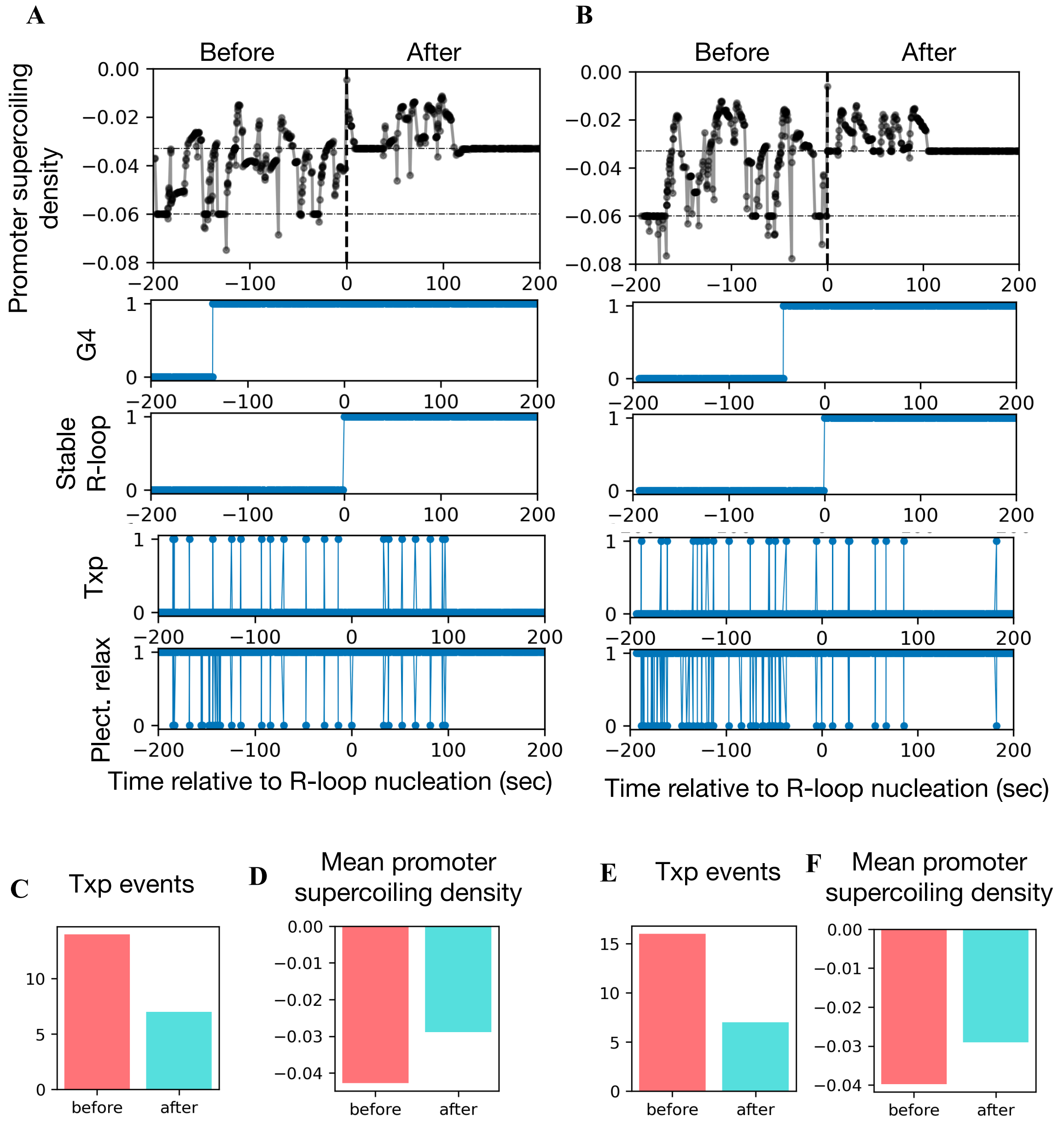

Supplementary Fig. 15: Sample simulation trajectories showing the connection between supercoiling and transcription initiation.

(A-B) Sample simulation trajectories for a PQS sequence, showing the promoter supercoiling density as a function of time. The origin of time is shifted to the nucleation of the stable R-loop state. The subpanels show the nucleation of the G4, stable R-loop, transcription events, and plectoneme relaxation events. (C) Number of transcription initiation events (number of peaks in the trajectory) and (D) mean promoter supercoiling density before and after the nucleation of the stable R-loop corresponding to the trajectory in (A). Similarly, (E) Number of transcription initiation events, and (F) Mean promoter supercoiling density before and after the nucleation of the stable R-loop corresponding to the trajectory in (B). One can clearly discern the lowering of promoter supercoiling and suppression of transcription initiation upon nucleating the stable R-loop, which is preceded by G4 nucleation.

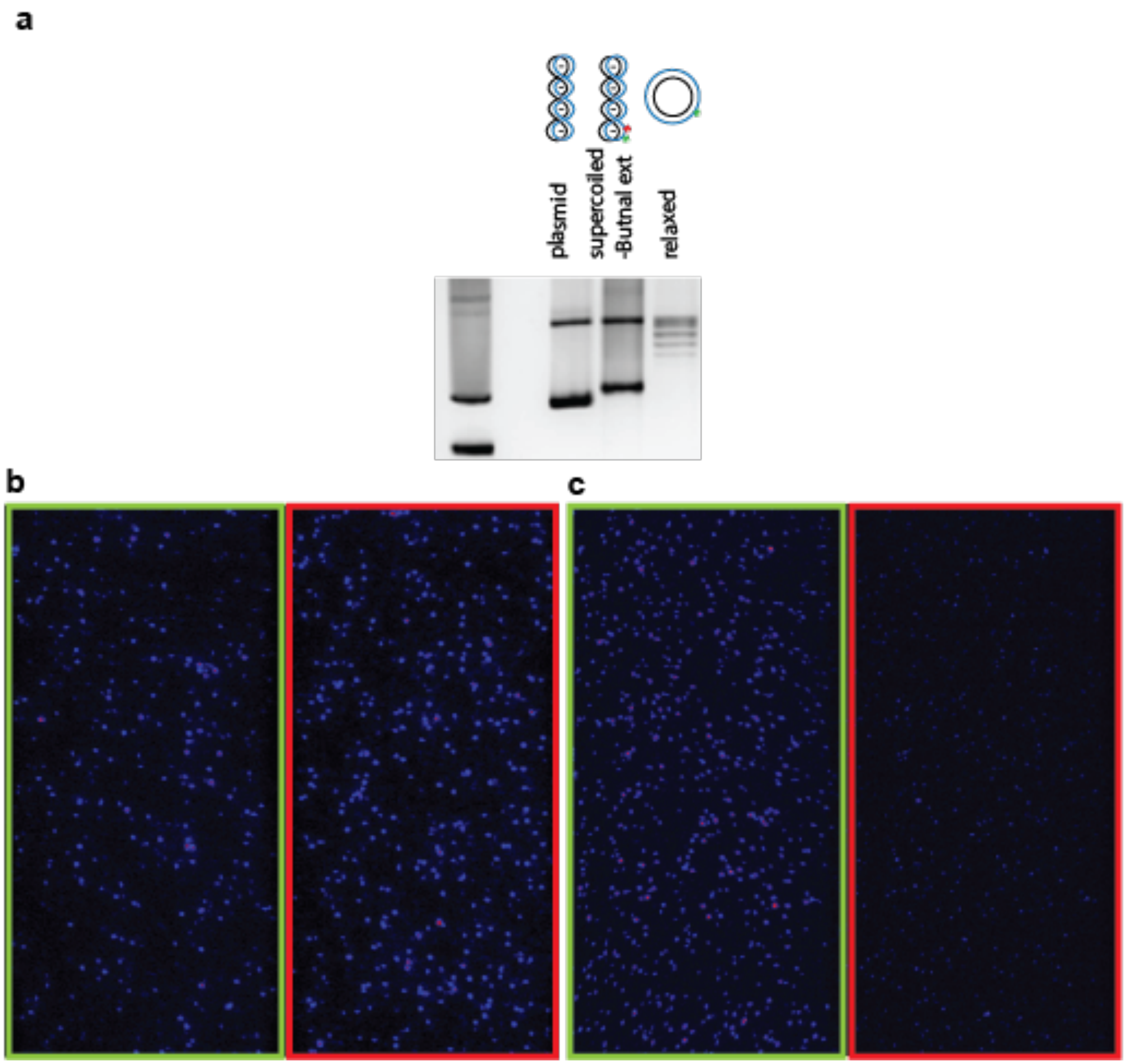

Supplementary Fig. 16: EtBr remains on ligated DNA without butanol extraction.

(a) In the absence of butanol extraction (second lane of the gel, supercoiled -Butanol ext), ligated DNA containing EtBr migrates higher on the agarose gel compared to non-treated plasmid. However, following butanol extraction, the supercoiled reconstituted construct migrates to the same position as the untreated plasmid, as shown in Figure 4b. (b) Single-molecule imaging reveals residual EtBr on the plasmid DNA, observed as a red signal upon 532 nm laser excitation, due to EtBr's broad emission spectrum. (c) After butanol extraction, these red signals disappear, confirming the effective removal of EtBr and resulting in the intended low FRET signal.

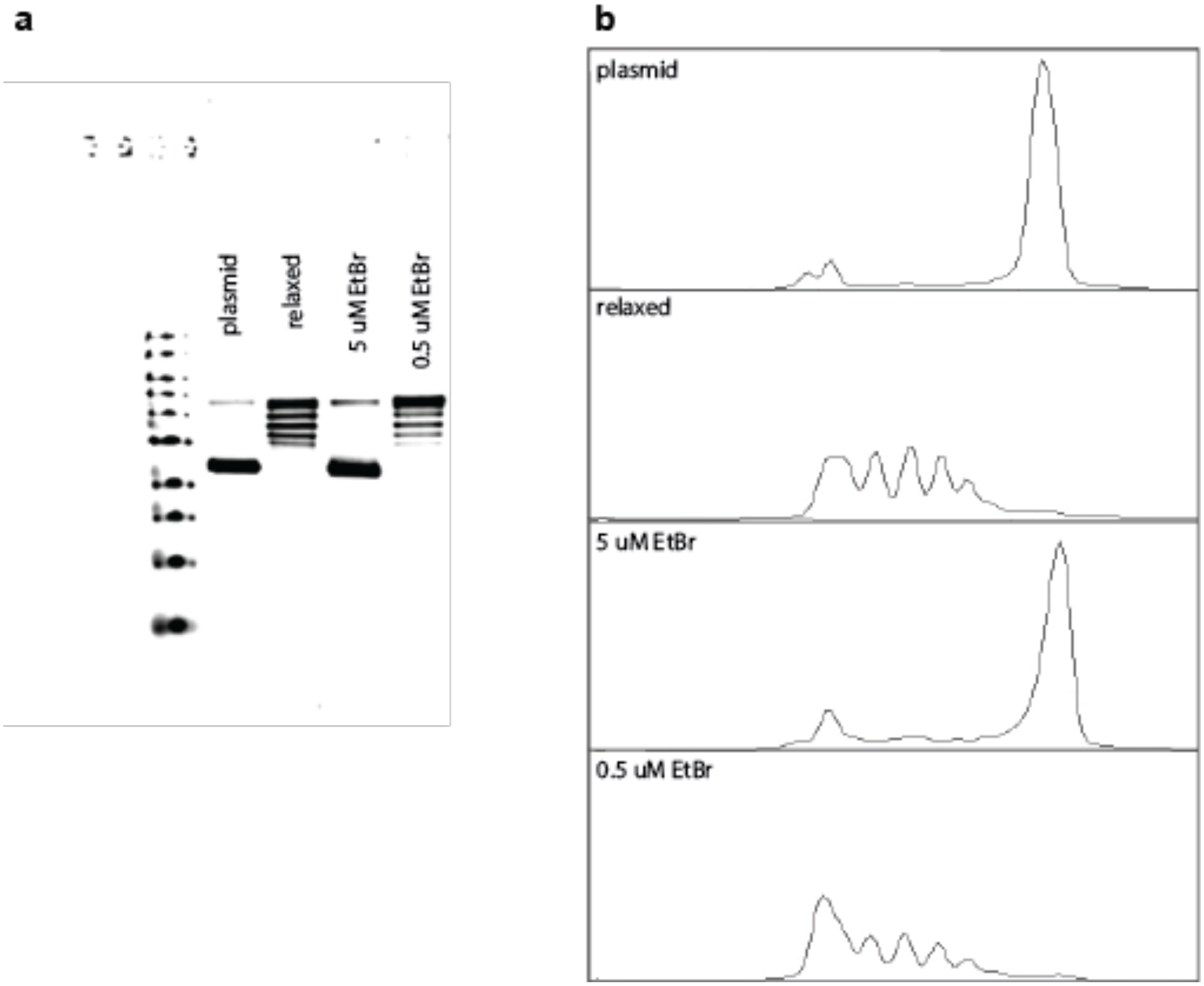

Supplementary Fig. 17: supercoiling density depends on EtBr concentration.

DNA treated with 5 μM EtBr (third lane) during the ligation process exhibits a supercoiling density similar to that of the non-modified plasmid. In contrast, DNA treated with a lower concentration of EtBr (0.5 μM) shows reduced supercoiling density, resulting in a more relaxed DNA structure. This confirms that supercoiling density is directly dependent on the concentration of EtBr as shown in Wang, Y. et al., *ACS Omega* (2019)[3].

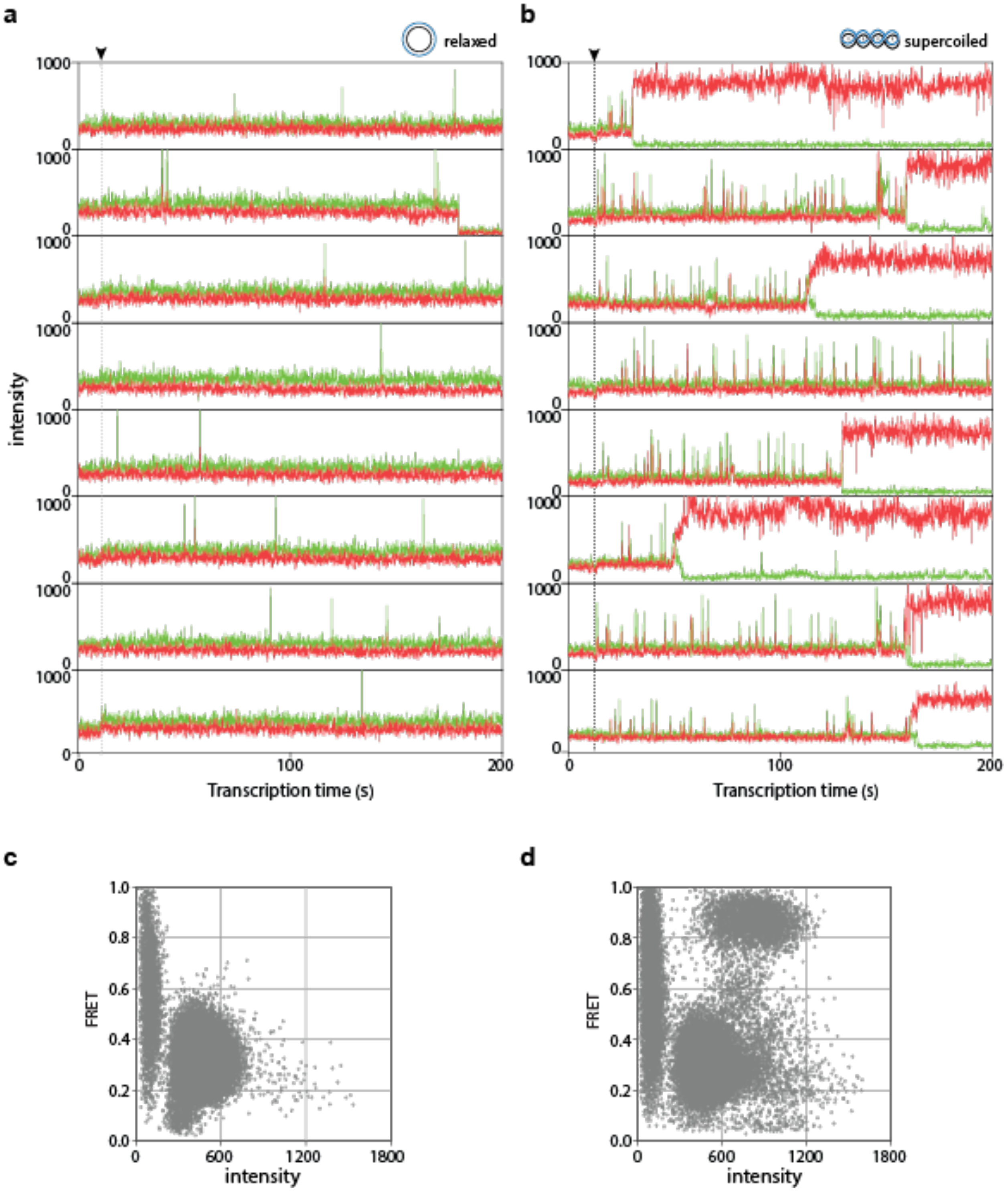

Supplementary Fig. 18: Representative single-molecule traces and intensity vs. FRET distribution for relaxed and supercoiled [FRET1] constructs.

(a and b) Representative single-molecule time traces of RNAP transcription for relaxed (a) and supercoiled (b) [FRET1] DNA constructs. Cy3 and Cy5 signals are shown in green and red, respectively. The dashed gray line indicates the time point when RNAP is introduced into the channel. In the relaxed [FRET1] construct (a), no FRET changes are observed, but short-lived PIFE signals are present. In the supercoiled [FRET1] construct (b), FRET transitions are observed, accompanied by short-lived PIFE peaks.

(c and d) Intensity vs. FRET distributions for accumulated traces of relaxed (c) and supercoiled (d) [FRET1] DNA constructs. The distributions are based on more than 50 molecules; exceeding this number results in dot density that obscures the clean distribution. In the relaxed construct (c), only populations with increased intensity and unchanged FRET values are observed. In contrast, the supercoiled construct (d) shows a high-FRET population (~0.9) with enhanced intensity and a mid-FRET population (~0.4–0.7) with increased intensity compared to the relaxed construct.

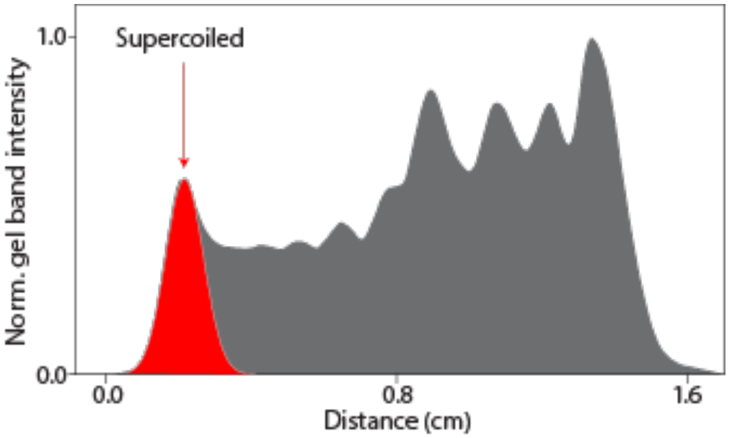

$$\text{Relaxed (\%)} = \left(\frac{\text{Total} - \text{supercoiled}}{\text{Total}}\right) \times 100$$

Supplementary Fig. 19: Gel band distribution showing the supercoiled area quantified by Gaussian fitting

The gel band intensities were obtained from gel images using Fiji and normalized to show relative distribution. The red-highlighted area represents the supercoiled DNA, which was fitted with a Gaussian function in Origin Pro 2018 to determine its area. The total area under the curve represents the combined intensities of all DNA topological states. The relaxed fraction was subsequently calculated as the difference between the total area and the supercoiled area, divided by the total area, as described in the Methods section.

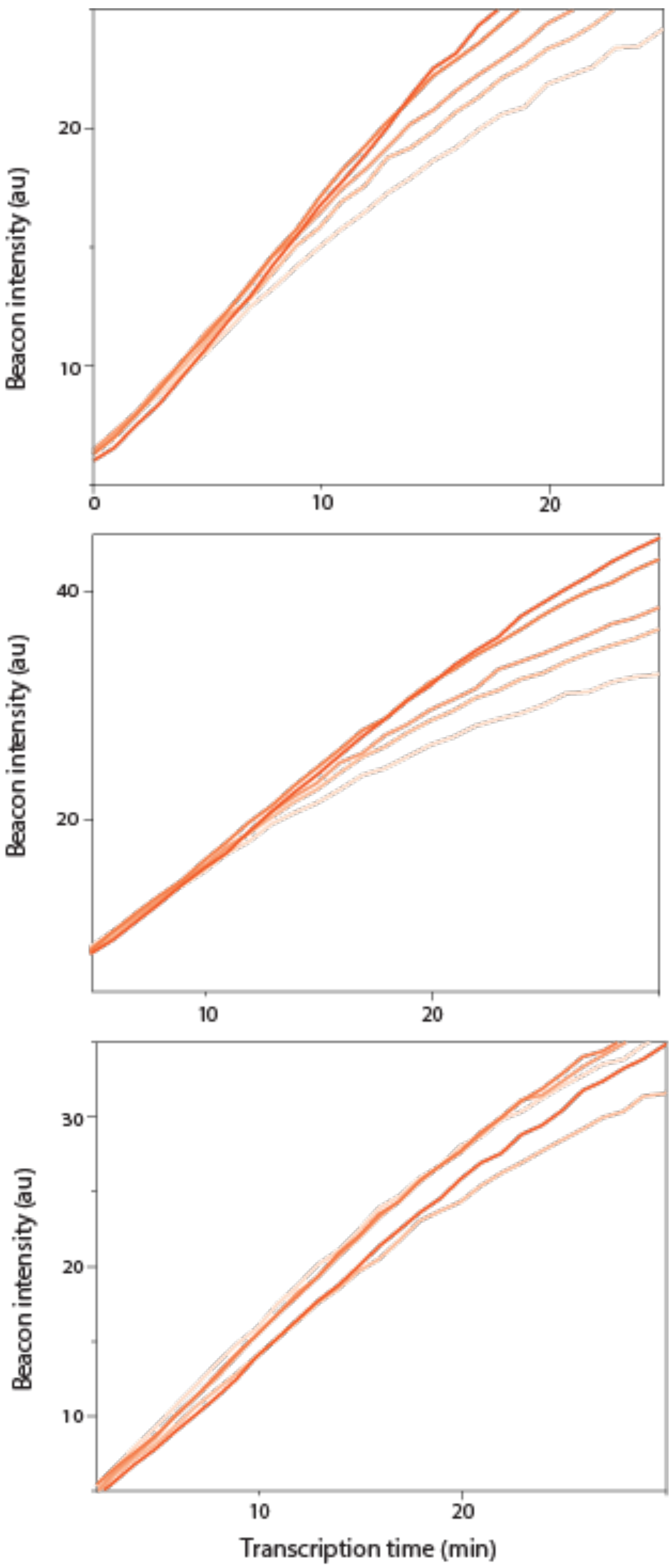

Supplementary Fig. 20: Linear region of transcription curves used for rate determination in Figure 3a.

This figure shows the initial linear region of transcription curves (up to approximately 20 minutes) used to assess transcription rates for different constructs[4]. These linear portions were selected based on the consistent initial increase in beacon intensity, allowing for a standardized comparison of transcription rates across different constructs.

Supplementary table 1: Sequences of DNA substrate in each assay.

**Item name**	**Sequence (5’ → 3’)**	**Substrate**
c-Myc	**GGGTGGGTAGGGTGGG**	PQS
T7 Promoter	TAATACGACTCACTATAGG	Promoter
rrrnB T1	CAAATAAAACGAAAGGCTCAGTCGAAAGACTGGGCCTTTCGTTTTATCTGTTGTTTGTCCGTGAACGCTCTCCTGAGTAGGACAAAT	terminator
Beacon probe	Cy3-TTCACCCTCTCCACGGAC-BHQ_2	Ensemble
sp10	AATTCGCGAATTAATACGACTCACTATAGGCAGAACATTT**GGGTGGGTAGGGTGGG**TT	Ensemble
sp15	AATTCGCGAATTAATACGACTCACTATAGGTCCGCATTTGGATTT**GGGTGGGTAGGGTGGG**TT	Ensemble
sp20	AATTCGCGAATTAATACGACTCACTATAGGTCCGCATTTGGAGCTACTTT**GGGTGGGTAGGGTGGG**TT	Ensemble
sp25	AATTCGCGAATTAATACGACTCACTATAGGGCTATTCGTTGCCTGTGATGGTATTT**GGGTGGGTAGGGTGGG**TT	Ensemble
sp30	AATTCGCGAATTAATACGACTCACTATAGGGGAATTGTGAGCGGATAACAATTCCCCTTT**GGGTGGGTAGGGTGGG**TT	Ensemble
sp35	AATTCGCGAATTAATACGACTCACTATAGGGGTAGCGAACGGGTTTGACACTTCACAGATAGTTTT**GGGTGGGTAGGGTGGG**TT	Ensemble
sp45	AATTCGCGAATTAATACGACTCACTATAGGTGTCCTGCGGTTACCCATGGCCTGTAATCCAGCTCGAGTCAATTT**GGGTGGGTAGGGTGGG**TT	Ensemble
sp60	AATTCGCGAATTAATACGACTCACTATAGGTTGAAGCGAAACGTTAACCGGAATCACTTGAGATACGAAACGCATGTGGAGACCAGGTTT**GGGTGGGTAGGGTGGG**TT	Ensemble
Control	AATTCGCGAATTAATACGACTCACTATAGGTGTCCTGCGGTTACCCATGCCATGTAATCCAGCTCGAGTCAATGAAGTC	Ensemble
T-PQS	AATTCGCGAATTAATACGACTCACTATAGGCCAATACAAAGGCTTCATCCTCACTCGAAA**CCCACCCATCCCACCC**AA	Ensemble

[FRET1]-NT-PQS-oligo1	Phos-TCAGCTTAATACGACTCACTATAGGCCAATACAAGAGCTTCATCCTCAGC/iAmMC6T/TT**GGGTG**	[FRET1]-NT-PQS
[FRET1]-NT-PQS-oligo2	Phos-**GGTAGGGTGGG**T/iAmMC6T/ATATGGTTGAAGGTAGTGGTAGTGGTCC	[FRET1]-NT-PQS
[FRET2]-NT-PQS-oligo1	Phos-TCAGCTTAATACGACTCAC/iAmMC6T/ATAGGCCAATACAAGAGCTTCATCC	[FRET2]-NT-PQS
[FRET2]-NT-PQS-oligo2	Phos-TGAGGA/iAmMC6T/GAAGCTCTTGTATTGGCCTATAGTGAGTCGTATTAAGC	[FRET2]-NT-PQS
[FRET1/2]-oligo3	Phos-TCAGCTTTTCCCAGTCAGTA/iBiodT/GTAGTAAAACGACTTCCTCAGCATTCGCGAACC	[FRET1/2]-NT-PQS or control
[FRET1]-Control-oligo1	Phos-TCAGCTTAATACGACTCACTATAGGCCAATACAAGAGCTTCATCCTCAGCAA/iAmMC6T/CCGTA	[FRET1]-Control
[FRET1]-Control-oligo2	Phos-TCAAG/iAmMC6T/CAATGAAGTCTTGAAGGTAGTGGTAGTGGTGATCC	[FRET1]-Control
[FRET2]-Control-oligo1	Phos-TCAGCTTAATACGACTCAC/iAmMC6T/ATAGGCCAATACAAGAGCTTCATCC	[FRET2]-Control
[FRET2]-Control-oligo2	Phos-TGAGGA/iAmMC6T/GAAGCTCTTGTATTGGCCTATAGTGAGTCGTATTAAGC	[FRET2]-Control

REFERENCE

1. Duquette, M. L., Handa, P., Vincent, J. A., Taylor, A. F. & Maizels, N. Intracellular transcription of G-rich DNAs induces formation of G-loops, novel structures containing G4 DNA. *Gene Dev* **18**, 1618–1629 (2004).

2. Zheng, K. *et al.* Detection of genomic G-quadruplexes in living cells using a small artificial protein. *Nucleic Acids Res* gkaa841- (2020) doi:10.1093/nar/gkaa841.

3. Wang, Y. *et al.* Kinetic Study of DNA Topoisomerases by Supercoiling-Dependent Fluorescence Quenching. *Acs Omega* **4**, 18413–18422 (2019).

4. Lee, C.-Y. *et al.* R-loop induced G-quadruplex in non-template promotes transcription by successive R-loop formation. *Nat Commun* **11**, 3392 (2020).
